# Should routine neonatal circumcision be a policy to prevent penile cancer? | *Opinion: No*

**DOI:** 10.1590/S1677-5538.IBJU.2017.01.04

**Published:** 2017

**Authors:** Dominic H. Tang, Philippe E. Spiess

**Affiliations:** 1 Department of Genitourinary Oncology, Moffitt Cancer Center, Tampa, Florida, USA

**Keywords:** prevention and control [Subheading], Circumcision, Male, Penile Neoplasms, Phimosis

Routine neonatal circumcision remains a controversial topic. The most recent Canadian Paediatric Society does not recommend routine circumcision of every newborn male (1). And although prior statements from the American Academy of Pediatrics recommended against circumcision, the most recent recommendation states that circumcision outweigh the risk and the procedure’s benefits justify it for families who want it (2). The benefits mentioned by the American Academy of Pediatrics included prevention of urinary tract infections, transmission of sexually transmitted infections, and penile cancer. Prevention of penile cancer may be related to increasing daily hygiene and decreasing sexually transmitted infections such as human papilloma virus (HPV) in circumcised males. However, with improvements in daily hygiene and sexually transmitted infection prevention strategies, neonatal circumcision may not be critical for the prevention of penile cancer, especially in western countries.

The association between lack of circumcision and penile cancer has been well documented (3). A meta-analysis found a strong protective effect of childhood circumcision on invasive penile cancer in 3 studies (OR 0.33; 95% 0.13-0.83) (3). However, in other studies when analyses were restricted to boys without a history of phimosis, the meta-analysis found that the protective effect of childhood circumcision on invasive disease no longer persisted (3). In addition, penile cancer continues to be one of the rarer malignancies in the world. In the United States where circumcision is prevalent, the rarity of disease is highlighted by its decreasing incidence. An analysis from the Surveillance, Epidemiology and End Results (SEER) database showed that the penile cancer incidence decreased to 0.58 per 100,000 in 1993-2002 from 0.84 per 100,000 in 1973-1982 in the United States (4). However, there is also a similar trend in countries with low circumcision rates such as Denmark and Finland (5, 6). In Denmark, penile cancer risk decreased from 1.15 per 100,000 in the 1940s to 0.82 per 100,000 in the late 1980s (5). Finland reported an incidence rate of 0.5 per 100,000 (6). Coupled with the rarity of penile cancer and conflicting evidence in the literature, it is hard to justify routine neonatal circumcision for all healthy males, including those without any preputial abnormalities, solely for the prevention of penile cancer.

Despite its rarity, there are several common factors that have been implicated to cause penile cancer. Factors resulting in phimosis, balanitis, and smegma have been associated with penile cancer in a meta-analysis (7). This has often been attributed to the lack of circumcision and poor hygiene as a major risk factor in developing these inflammatory conditions. However, with improved daily hygiene, these conditions can still be combatted even with a lack of circumcision. For example, in the Denmark population where there is only a 2% circumcision rate, the incidence of penile cancer was shown to be steadily declining, likely coinciding with an increase in indoor bathrooms and improved hygiene (5). However, it’s notable that the most recent study of the same population found an increasing rate of penile cancer from 1978 to 2008 (8). This study reported an average annual percentage change of 0.8% in incidence resulting in an increase in incidence to 1.3 per 100,000 men in 2008. Although this study did not contain data regarding HPV status, the prevalence of HPV in Denmark has also been found to be as high as 33-45% of men in several reports (9, 10). In addition, several studies have shown an increase in HPV-associated cancers in Denmark over a similar time period (11, 12). Therefore, it is unlikely that an increase in penile cancer incidence can solely be explained by a low circumcision rate. Rather, it may be the HPV associated penile carcinogenesis that plays a larger factor in disease incidence in this cohort.

As a driving factor for penile carcinogenesis, the prevalence of HPV induced penile cancer has been shown to be approximately 50% of penile malignancies worldwide (13). Circumcision has also been known to play a role in the prevention of sexually transmitted diseases such as HPV. In addition, the association between circumcision and reduced risk of penile HPV has been well documented in several randomized clinical trials and meta-analyses (14-16). However, it’s important to note that these data represent an exclusively adult cohort as there are no studies on the association between infant circumcision and risk for sexually transmitted disease. What can be justified based on this data is the counseling of the benefits of circumcision to an adult male to help reduce his risk of HPV related infection and disease, in addition to modifiable behaviors such as condom use.

Prevention of HPV infection and subsequent penile cancer risk can also be accomplished with vaccines. There are two prophylactic HPV vaccines have been developed that can play an important role in the prevention of HPV transmission. This includes the quadrivalent and 9-valent HPV vaccines (Gardasil and Gardasil 9) that have been licensed for use in females and males (17). The efficacy of these vaccines has been demonstrated in recent studies among HPV-negative men and women (18, 19). Vaccine administration is approved for males aged 9 through 26 years (17) and can be given prior to the counseling of undergoing circumcision later in life. Therefore, it may be reasonable to delay circumcision as a neonate until the patient approaches sexual maturity. This can prevent the need for routine circumcision for all male neonates and reserve circumcision to only those who develop risk factors such as high risk sexual practices or abnormalities such as phimosis or balanitis.

Penile carcinoma can be preventable through advocating daily hygiene and HPV prevention. Although circumcision can help reduce risk factors for penile cancer development, this does not necessarily warrant a requirement for circumcision as a neonate given the rarity of disease and alternative strategies in prevention. Through improved efforts in modifiable behaviors and implementation of HPV vaccination, this can curb penile cancer risk until the patient can make an informed decision regarding circumcision later in life.
